# Supplementary material for: Correlations between oral Pre-Exposure Prophylaxis (PrEP) initiations and policies that enable the use of PrEP to address HIV globally
Source: PLOS Glob Public Health. 2022 Dec 7;2(12):e0001202. doi: 10.1371/journal.pgph.0001202 (PMC10021197; doi:10.1371/journal.pgph.0001202)
Supplement: Attachment — Submitted filename: PLOS Global Public Health Response to Reviewers.docx [file pgph.0001202.s001.docx]

**Response to Reviewers: Reviewer Comments & Author Responses**

Note: the references pages and line numbers refer to the original manuscript submitted prior to review. As formatting line numbers and track changes in Microsoft Word were incompatible, line numbering on the revised manuscript is at times misaligned.

Comment: Keywords: Suggestion: consult the Decs and adjust the words...

(Page 1, Keywords section)

Original: “HIV Prevention; oral pre-exposure prophylaxis; HIV PrEP; PrEP uptake; policy environment; HIV self-testing; Age of consent; PrEP eligibility; PrEP regulatory

approval; PrEP guidelines”

Revised: “***HIV***, HIV Prevention; ***pre-exposure prophylaxis***; oral pre-exposure prophylaxis; HIV PrEP; PrEP uptake; policy environment; HIVself-testing; Age of consent; PrEP eligibility; PrEP regulatory approval; PrEP guidelines”

Author Response: Thank you for this suggestion. We have used the [DeCS/MeSH Health Sciences](https://decs.bvsalud.org/en/) Descriptors to incorporate additional keywords into our manuscript.

Comment: Title: Suggestion: change the more appropriate title: "Correlations between Initiation and Oral Preparation Policies that enable the use of pre-exposure prophylaxis to HIV worldwide"

(Page 2, line 1)

Original: “Policies influence practice: national policies and their correlations with oral prep initiation rates”

Revised: “Correlations Between Oral Pre-Exposure Prophylaxis (PrEP) Initiations and Policies that Enable the Use of PrEP to Address HIV Globally”

Author Response: We accepted this suggestion and have further edited the title from “Policies influence practice: national policies and their correlations with oral prep initiation rates” to “Correlations between Oral Pre-Exposure Prophylaxis (PrEP) Initiations and Policies that Enable the Use of PrEP to Address HIV Globally.”

Comment: Very extensive introduction. Suggestion: summarize. Describe: which continents evaluated, in the world. What are the study variables? How long the research took and how it was carried out (tools used). In conclusion: include that public health policies, in this sense, should be reanalyzed and/or implemented.

(Page 1, line 19)

Original: “Policies facilitating access to HIV prevention services can foster enabling environments for service uptake by removing barriers for those in need. This is especially so when evidence indicates that oral pre-exposure prophylaxis (PrEP) significantly reduces HIV incidence, HIV self-testing is acceptable and accurate, lowered age of consent to HIV services are conducive to increased youth access to PrEP, and PrEP uptake increases with policy support for broad PrEP eligibility. We collected data from the HIV Policy Lab and AVAC’s Global PrEP Tracker and conducted linear regression and correlation analyses via STATA to examine relationships amongst national oral PrEP regulatory approval and eligibility criteria, HIV self-testing, lowered age of consent, and national cumulative oral PrEP initiations. Of 194 countries tracked by the HIV Policy Lab, 92 (47.4%) have adopted HIV self-testing policies, 44 (22.7%) have adopted policies authorizing lowered age of consent, and 93 (47.9%) have either fully or partially adopted policies authorizing PrEP eligibility, of which only 54 (27.8%) have fully adopted. Of all 194 countries, only 44 (22.7%) have co-adopted HIV self-testing 32 and PrEP eligibility policies, and 15 (7.7%) have co-adopted lowered age of consent and PrEP eligibility policies . Only 13 (6.7%) have adopted all three policies. Analyses conducted for the 94 countries tracked by both the HIV Policy Lab and the Global PrEP Tracker revealed that there is a significant and positive relationship between estimated number of cumulative individuals initiated on oral PrEP and adoption of HIV self-testing policies (p=0.01; r=0.26), adoption of lowered age of consent policies (p=0.01, r=0.25), and adoption of PrEP eligibility policies (p=0.01, r=0.26). Despite the significant relationship between these policies and oral PrEP uptake, many countries have yet to adopt any or all three policies. Stronger advocacy efforts towards approving policies that enshrine and enable access to HIV prevention are necessary.”

Revised: “Policies facilitating access to HIV prevention services, specifically for pre-exposure prophylaxis (PrEP), canfoster enabling environments for service uptake. This analysis aims to establish whether policies enabling broad PrEP eligibility, HIV self-testing, and lowered age of consent to HIV testing and treatment services are correlated with PrEP uptake. Ages of consent vary by country, therefore this analysis focused on how age of consent policies, in general, affect adolescent PrEP uptake. Data was collected from the HIV Policy Lab and AVAC’s Global PrEPTracker, a database of approximately 180 PrEP projects operating across 95 countries, and linear regression and correlation analyses were conducted via STATA to examine relationships amongst national oral PrEPeligibility, HIV self-testing, lowered age of consent, and national cumulative oral PrEP initiations, as of December 2021. Of all 194 countries tracked by the HIV Policy Lab, only about 7% have adopted all three policies. Less than 50% of these countries have adopted either HIV self-testing policies, lowered age of consent policies, or PrEP eligibility policies. Of the 54 countries that have fully adopted PrEP eligibility policies, less than 30% have co-adopted HIV self-testing or lowered age of consent policies. About 30% of these 194 countries have yet to adopt any policy at all, where about 14% have indicated “unavailable” information for at least one of the policies. Analyses conducted for the 91 countries tracked by both the HIV Policy Lab and the Global PrEP Tracker revealed a significant and positive relationship between cumulative individuals initiated on oral PrEP and adoption of HIV self-testing policies (p=0.01, r=0.26), lowered age of consent policies (p=0.01, r=0.25), and PrEP eligibility policies (p=0.01, r=0.26). Stronger advocacy efforts towards approving public health policies, such as those outlined in our analysis, that enshrine and enable access to HIV prevention are necessary.”

Author Response: Thank you for this suggestion. This comment was included in the Abstract section and hence we have made changes to the Abstract to include information on study variables by highlighting the aim of this study. The study variables are the adoption status of each of the three policies under investigation and the cumulative national oral PrEP initiations recorded for a country. We specified that we included information on 194 countries; as such the analysis was global. We also highlighted that the research was carried out using STATA calculations. We added a concluding sentence where we indicated that advocacy efforts need to be strengthened to enshrine public health policies that enable access to HIV prevention services. In addition, we summarized our findings to further shorten the abstract.

Comment: And the keywords? Sugestion: include

(Page 1, line 19)

Author Response: Thank you for this suggestion. To accommodate the 300 word limit on the abstract and to include the details in the abstract that reviewers suggested to include, we have kept the keywords in the Keywords Section for this paper.

Comment: If: "Only 13 (6.7%) have adopted all three policies..." How: "many countries have yet to adopt any or all three policies"???? Controversial! Suggestion: review here and in the Results section!

(Page 2, line 33-40)

Original: “Of 194 countries tracked by the HIV Policy Lab, 92 (47.4%) have adopted HIV self-testing policies, 44 (22.7%) have adopted policies authorizing lowered age of consent, and 93 (47.9%) have either fully or partially adopted policies authorizing PrEP eligibility, of which only 54 (27.8%) have fully adopted. Of all 194 countries, only 44 (22.7%) have co-adopted HIV self-testing 32 and PrEP eligibility policies, and 15 (7.7%) have co-adopted lowered age of consent and PrEP eligibility policies . Only 13 (6.7%) have adopted all three policies. Analyses conducted for the 94 countries tracked by both the HIV Policy Lab and the Global PrEP Tracker revealed that there is a significant and positive relationship between estimated number of cumulative individuals initiated on oral PrEP and adoption of HIV self-testing policies (p=0.01; r=0.26), adoption of lowered age of consent policies (p=0.01, r=0.25), and adoption of PrEP eligibility policies (p=0.01, r=0.26). Despite the significant relationship between these policies and oral PrEP uptake, many countries have yet to adopt any or all three policies.”

Revised: “Of all 194 countries tracked by the HIV Policy Lab, only about 7% have adopted all three policies (HIV self-testing, lowered age of consent, and PrEP eligibility policies). Less than 50% have adopted at least one of these policies. Of the 54 countries that have fully adopted PrEP eligibility policies, less than 30% have co-adopted HIV self-testing or lowered age of consent policies. About 30% of these 194 countries have yet to adopt any of these policies, of which about 14% have indicated information is “unavailable” for at least one of the policies. Analyses conducted for the 91 countries tracked by both the HIV Policy Lab and the Global PrEP Tracker revealed a significant and positive relationship between cumulative individuals initiated on oral PrEP and adoption of HIV self-testing policies (p=0.01, r=0.26), lowered age of consent policies (p=0.01, r=0.25), and PrEP eligibility policies (p=0.01, r=0.26).”

Author Response: Thank you for pointing this out. Of 194 countries tracked by the HIV Policy Lab, only 13 (7%) have adopted all three policies. We have moved this finding upfront to underscore how few countries have adopted these three policies and clarified how many countries have yet to adopt any policies at all (59 countries), of which 28 countries indicated information was “unavailable” as the status for at least one of the policies. Those with a partially adopted status for any policy counted as having been adopted; this information has also been added to the results section as suggested. The abstract was shortened per reviewers’ suggestions and hence this information has been summarized there.

Comment: What about Public Health Policies???

(Page 2, line 41)

Original: “Stronger advocacy efforts towards approving ***policies*** that enshrine and enable access to HIV prevention are necessary.”

Revised: “Stronger advocacy efforts towards approving ***public health*** ***policies, such as those outlined in our analysis,*** that enshrine and enable access to HIV prevention are necessary.”

Author Response: Thank you for helping us further clarify this sentence. We did indeed intend to say that we need more advocacy efforts to approve public health policies that enshrine and enable access to HIV prevention such as the three policies we cover in our analysis (HIV self-testing, lowered age of consent, PrEP eligibility).

Comment: Suggestion: delete word

(Page 2, line 45)

Original: “In 2020, there were an estimated 1.5 million new HIV infections globally, bringing the number of people living with HIV to approximately ***37.7 million*** ***worldwide***.”

Revised: “In 2020, there were an estimated 1.5 million new HIV infections globally, bringing the number of people living with HIV to approximately ***37.7 million***.”

Author Response: This word was repetitive and has been deleted.

Comment: The focus of the study was which age group? Suggestion: define and insert which age group is defined in the Abstract, here in the Introduction focus on the chosen age(s) and in the Methodology make it clear as well.

(Page 4, lines 80-96)

Original: “Based on extensive analysis, UNICEF found that it is crucial to lower the age of consent laws for accessing HIV and sexual reproductive health services to ensure that all adolescents who need services can access them, which can be conducive to improving HIV prevention outcomes in this group [17]. Adolescents represent a growing proportion of individuals living with HIV worldwide, with UNICEF reporting that in 2020, approximately 1.75 million adolescents between 10 and 19 were living with HIV globally [18]. In 2020, approximately 400,000 adolescents and young adults between the ages of 10 and 24 were newly infected with HIV [18]. As of July 2021, data suggests that only 25% of adolescent girls and 17% of adolescent boys aged 15-19 had been tested for HIV in the past 12 months in Eastern and Southern Africa, the region most severely impacted by HIV [18]. Testing rates for adolescents and young adults are even lower in Western Africa and South Asia [18]. Lowering age of consent laws for accessing HIV and sexual reproductive health services can help reduce the multiple barriers, including structural barriers, to access and adherence faced by adolescents and young adults compared to adults [19]. Furthermore, legal hurdles for those below the age of legal adulthood, lack of clarity surrounding the need for parental consent and parameters conducive to adolescent consent, and other legal inconsistencies related to HIV health services further impede adolescents’ access to PrEP [19].”

Revised: “Based on extensive analysis, UNICEF found that it is crucial to lower the age of consent laws for accessing HIV and sexual reproductive health services to ensure that all adolescents who need services can access them, which can be conducive to improving HIV prevention outcomes in this group [17]. Adolescents represent a growing proportion of individuals living with HIV worldwide, with UNICEF reporting that in 2020, approximately 1.75 million adolescents between 10 and 19 were living with HIV globally [18]. In 2020, approximately 400,000 adolescents and young adults between the ages of 10 and 24 were newly infected with HIV [18]. As of July 2021, data suggests that only 25% of adolescent girls and 17% of adolescent boys aged 15-19 had been tested for HIV in the past 12 months in Eastern and Southern Africa, the region most severely impacted by HIV [18]. Testing rates for adolescents and young adults are even lower in Western Africa and South Asia [18]. Lowering age of consent laws for accessing HIV and sexual reproductive health services can help reduce the multiple barriers, including structural barriers, to access and adherence faced by adolescents and young adults compared to adults [19]. Furthermore, legal hurdles for those below the age of legal adulthood, lack of clarity surrounding the need for parental consent and parameters conducive to adolescent consent, and other legal inconsistencies related to HIV health services further impede adolescents’ access to PrEP [19]. ***Age of consent for HIV prevention services varies by country based on definitions established by national governments; policies can apply to individuals 10 years and older. This analysis sought to understand the effect of lowered age of consent policies on adolescent access to PrEP.”***

Author Response: Age of consent varies by country as it is defined by national governments. Hence, we did not focus on a particular age group. Our aim was to understand the general influence of age of consent on PrEP uptake amongst adolescents. Our literature review suggests that the more restrictive the age of consent, i.e. the older the age of consent, the less likely adolescents are to access PrEP. We have clarified this in the abstract, introduction, and methodology.

Comment: This whole part is characterized more as Discussion of the manuscript. Suggestion: remove and include in the Discussion section. And here, if you want to keep the information, summarize it well and objectively, putting only the source.

(Page 5, lines 98-110)

Original in Introduction: “Despite these barriers, evidence suggests that youth, including 98 adolescent girls and young women (AGYW) and young members of key populations are motivated to access PrEP. A study conducted with 825 AGYW in Malawi found that PrEP interest was high among all perceived HIV risk levels, with the odds of showing interest in PrEP being more than doubled in AGYW

reporting high perceived risk of HIV acquisition [20]. A study in Malawi surveying 2,089 adolescents, ages 10-19, and caregivers showed that 82% of adolescents and 87% of caregivers were interested in allowing adolescents access to PrEP [21]. Another study focused on 200 adolescent men who have sex with men (MSM) and transgender women (TGW) PrEP users, ages 15-19, found that at least 50% of participants were motivated to retain PrEP use 6 months after intervention due to the presence of youth-friendly services [22]. Such services were made possible by Thailand’s policies allowing adolescents ages 13-18 access to HIV and STI testing and treatment without parental consent or approval of parental consent exemption by an ethics committee [22]. This highlights that policies accommodating adolescent consent are crucial.”

Revised in Discussion: “This supports findings from studies suggesting that young people are motivated to access PrEP. For instance, studies have found that up to 62% of AGYW in Malawi with no perceived risk of HIV acquisition, 69% with low perceived risk, and 78% with high perceived risk are motivated to access PrEP [20]. A study conducted with 825 AGYW in Malawi found that PrEP interest was high among all perceived HIV risk levels, with the odds of showing interest in PrEP being more than doubled in AGYW reporting high perceived risk of HIV acquisition [20]. Another study in Malawi surveying 2,089 adolescents, ages 10-19, and caregivers showed that 82% of adolescents and87% of caregivers were interested in allowing adolescents access to PrEP [21]. A study in Thailand focused on 200 adolescent men who have sex with men (MSM) and transgenderwomen (TGW) PrEP users, ages 15-19, found that at least 50% of participants were motivated toretain PrEP use six months after intervention due to the presence of youth-friendly services [22].Such services were the results of Thailand’s policies allowing adolescents ages 13-18 access toHIV and STI testing and treatment without parental consent or approval of parental consentexemption by an ethics committee [22]. A significant and positive correlation between countries authorizing lowered age of consent to HIV testing and treatment and cumulative number of PrEP initiations also supports findings from studies that highlight youth perspectives on access to PrEP in the context of parental consent. ”

Author Response: Thank you for this suggestion. This paragraph has been moved to and integrated into the Discussion section.

Comment: Suggestion: insert this objective in the Summary.

(Pages 5-6, lines 117-120)

Original Abstract: “Policies facilitating access to HIV prevention services can foster enabling environments for service uptake by removing barriers for those in need. ***This is especially so when evidence indicates that oral pre-exposure prophylaxis (PrEP) significantly reduces HIV incidence, HIV self testing is acceptable and accurate, lowered age of consent to HIV services are conducive to increased youth access to PrEP, and PrEP uptake increases with policy support for broad PrEP eligibility.”***

Revised Abstract: “Policies facilitating access to HIV prevention services, specifically for pre-exposure prophylaxis (PrEP), canfoster enabling environments for service uptake. ***This analysis aims to establish whether policies enabling broad PrEP eligibility, HIV self-testing, and lowered age of consent to HIV testing and treatment services are correlated with PrEP uptake.”***

Author Response: We agree with this suggestion and have added a sentence highlighting the aim of our study into our abstract.

Comment: That's methodology. Suggestion: remove and insert in the Methodology section.

(Pages 5-6, lines 117-120)

Original Materials and Methods: “We used data from the HIV Policy Lab and AVAC’s Global PrEP Tracker, a comprehensive database of approximately 180 ongoing PrEP projects and programs operating across 95 countries. We collected data on policies authorizing eligibility for oral PrEP, self-testing and lowered age of consent to HIV testing and treatment in 194 countries from the HIV Policy Lab.”

Revised Materials and Methods: “***Understanding the importance of creating a conducive and enabling environment to support PrEP uptake and access to HIV prevention services, the aim of this analysis was to establish whether policies authorizing broad PrEP eligibility, HIV self-testing, and lowered age of consent to HIV testing and treatment services are correlated with PrEP uptake.*** As national governments set different ages of consent for access to HIV testing and treatment services across countries, we will focus on how age of consent, in general, affects adolescent uptake of PrEP rather than analyzing age of consent policies for particular countries. Study variables included the adoption status of the above three policies and cumulative PrEP initiations for each of 194 countries. We used data from the HIV Policy Lab and AVAC’s Global PrEP Tracker, a comprehensive database of approximately 180 ongoing PrEP projects and programs operating across 95 countries. We collected data on policies authorizing eligibility for oral PrEP, self-testing and lowered age of consent to HIV testing and treatment in 194 countries from the HIV Policy Lab, as of December 2021.”

Author Response: We agree and have moved the sentence highlighting the analytical aim of this study from the end of the Introduction section to the beginning of the Materials and Methods section.

Comment: To serve as a scientific basis for other studies... Suggestion: detail the methodology, include study variables, time, sources…

(Page 6, line 122)

Original: “We used data from the HIV Policy Lab and AVAC’s Global PrEP Tracker, a comprehensive database of approximately 180 ongoing PrEP projects and programs operating across 95 countries. We collected data on policies authorizing eligibility for oral PrEP, self-testing and lowered age of consent to HIV testing and treatment in 194 countries from the HIV Policy Lab. Each country reported a non-numeric policy status, which was numerically coded for analysis as follows: unavailable was coded as 0, not adopted was coded as 1, planning to submit was coded as 2, under review was coded as 3, partially adopted was coded as 4, and adopted was coded as 5. We also collected data on the cumulative number of PrEP initiations as of December 2021 for each of the 95 countries in AVAC’s Global PrEP Tracker. We performed Linear Regression and Correlations and Covariances analyses using STATA (STATA/SE 17.0) to evaluate relationships amongst national policies that authorize PrEP eligibility, HIV self-testing and lowered age of consent to HIV testing and treatment, and their correlation with oral PrEP initiations. Threshold of significance was p=0.05.”

Revised: “Understanding the importance of creating a conducive and enabling environment to support PrEP uptake and access to HIV prevention services, the aim of this analysis was to establish whether policies authorizing broad PrEP eligibility, HIV self-testing, and lowered age of consent to HIV testing and treatment services are correlated with PrEP uptake. As national governments set different ages of consent for access to HIV testing and treatment services across countries, we will focus on how age of consent, in general, affects adolescent uptake of PrEP rather than analyzing age of consent policies for particular countries. ***Study variables included the adoption status of the above three policies and cumulative PrEP initiations for each of 194 countries. We used data from the HIV Policy Lab and AVAC’s Global PrEP Tracker, a comprehensive database of approximately 180 ongoing PrEP projects and programs operating across 95 countries.*** We collected data on policies authorizing eligibility for oral PrEP, self-testing and lowered age of consent to HIV testing and treatment in 194 countries from the HIV Policy Lab, ***as of December 2021***. Each country reported a non-numeric policy status, which was numerically coded for analysis as follows: unavailable was coded as 0, not adopted was coded as 1, planning to submit was coded as 2, under review was coded as 3, partially adopted was coded as 4, and adopted was coded as 5. We also collected data on the cumulative number of PrEP initiations as of December 2021 for each of the 95 countries in AVAC’s Global PrEP Tracker. We performed Linear Regression and Correlations and Covariances analyses using STATA (STATA/SE 17.0) to evaluate relationships amongst national policies that authorize PrEP eligibility, HIV self-testing and lowered age of consent to HIV testing and treatment, and their correlation with oral PrEP initiations. Threshold of significance was p=0.05.”

Author Response: Thank you for this suggestion. Study variables, time, and sources have been clarified in the Materials and Methods section. Study variables include the adoption status of HIV self-testing, broad PrEP eligibility, and age of consent policies, and cumulative PrEP initiations for each of the 194 countries under analysis. This analysis was conducted as a cross-sectional analysis of data available for the policy adoption statuses and cumulative PrEP initiation rates for 194 countries as of December 2021. Sources have already been specified (HIV Policy Lab and AVAC’s Global PrEP Tracker) and have not been changed.

Comment: Attention to Scientific Writing when writing articles. Suggestion: review Scientific Writing throughout the manuscript!

(Page 6, lines 123-131)

Original: “We used data from the HIV Policy Lab and AVAC’s Global PrEP Tracker, a comprehensive database of approximately 180 ongoing PrEP projects and programs operating across 95 countries. ***We collected data*** on policies authorizing eligibility for oral PrEP, self-testing and lowered age of consent to HIV testing and treatment in 194 countries from the HIV Policy Lab. Each country reported a non-numeric policy status, which was numerically coded for analysis as follows: unavailable was coded as 0, not adopted was coded as 1, planning to submit was coded as 2, under review was coded as 3, partially adopted was coded as 4, and adopted was coded as 5. ***We also*** collected data on the cumulative number of PrEP initiations as of December 2021 for each of the 95 countries in AVAC’s Global PrEP Tracker. ***We performed Linear Regression and Correlations and Covariances analyses*** using STATA (STATA/SE 17.0) to evaluate relationships amongst national policies that authorize PrEP eligibility, HIV self-testing and lowered age of consent to HIV testing and treatment, and their correlation with oral PrEP initiations. Threshold of significance was p=0.05.”

Revised: “Understanding the importance of creating a conducive and enabling environment to support PrEP uptake and access to HIV prevention services, the ***aim of this analysis*** was to establish whether policies authorizing broad PrEP eligibility, HIV self-testing, and lowered age of consent to HIV testing and treatment services are correlated with PrEP uptake. As national governments set different ages of consent for access to HIV testing and treatment services across countries, ***this study*** will focus on how age of consent, in general, affects adolescent uptake of PrEP rather than analyzing age of consent policies for particular countries. ***Study variables*** included the adoption status of the above three policies and cumulative PrEP initiations for each of 194 countries. ***This study*** used data from the HIV Policy Lab and AVAC’sGlobal PrEP Tracker, a comprehensive database of approximately 180 ongoing PrEP projects and programs operating across 95 countries. ***Data was collected*** on policies authorizing eligibility for oral PrEP, self-testing and lowered age of consent to HIV testing and treatment in 194 countries from the HIV Policy Lab, as of December 2021. Each country reported a non-numeric policy status, which was numerically coded for analysis as follows: unavailable was coded as 0, not adopted was coded as 1, planning to submit was coded as 2, under review was coded as 3, partially adopted was coded as 4, and adopted was coded as 5. ***Data was also collected*** on the cumulative number of PrEP initiations as of December 2021 for each of the 95 countries in AVAC’s Global PrEP Tracker. ***Linear Regression and Correlations and Covariances analyses were performed*** using STATA (STATA/SE 17.0) to evaluate relationships amongst national policies that authorize PrEP eligibility, HIV self-testing and lowered age of consent to HIV testing and treatment, and their correlation with oral PrEP initiations. Threshold of significance was p=0.05.”

Author Response: We have rewritten the Materials and Methods section in third person to accommodate scientific writing. We have also addressed scientific writing throughout the manuscript as suggested, including in the Introduction, Materials and Methods, and Discussion sections.

Comment: Suggestion: insert information in the Summary

(Page 6, lines 123-125)

Original Abstract: We collected data from the HIV Policy Lab and AVAC’s Global PrEP Tracker and conducted linear regression and correlation analyses via STATA to examine relationships amongst national oral PrEP regulatory approval and eligibility criteria, HIV self-testing, lowered age of consent, and national cumulative oral PrEP initiations.

Revised Abstract: “Data was collected from the HIV Policy Lab and AVAC’s Global PrEP Tracker, ***a database of approximately 180 PrEP projects operating across 95 countries,*** and linear regression and correlation analyses were conducted viaSTATA to examine relationships amongst national oral PrEP eligibility, HIV self-testing, lowered age of consent,and national cumulative oral PrEP initiations.”

Author Response: We agree that information about data sources used is important and have included it in the Abstract.

Comment: Suggestion: Graphs illustrate briefly and objectively, however, considering the number and scope of information, there is a need to build at least one very informative table that includes all the information found. Mainly to show the reader, in detail, how it was carried out and what findings the study reached through linear regression and correlation analyzes.

(Page 6, line 137)

Author Response: We agree with this suggestion and have added two tables. Table 1 depicts the number of countries that have a particular status for each policy and Table 2 depicts the significance and correlation values for each policy status with the cumulative number of PrEP initiations.

Comment: How many countries in total? Year?? Suggestion: improve figure titles…

(Pages 7-8, lines 154, 158, 162)

Original: “Fig 1. Percent of Countries by Adoption Status of HIV Self Testing Policies. 46.9% of countries have adopted policies authorizing HIV Self-Testing in-country, 50.0% have not adopted, and information is unavailable for 3.1% of countries.

Fig 2. Percent of Countries by Adoption Status of Age of Consent Policies. 22.4% of countries have adopted policies authorizing lowered age of consent to HIV testing and treatment services in-country, 57.7% have not adopted, and information is unavailable for 19.9% of countries.

Fig 3. Percent of Countries by Adoption Status of PrEP Eligibility 162 Policies. 27.6% of countries 163 have adopted policies authorizing PrEP approval in-country, 15.3% have partially adopted, 164 37.2% have not adopted, and information is unavailable for 19.9% of countries.”

Revised: “Fig 1. Percent of ***194*** Countries by Adoption Status of HIV Self Testing Policies***, Dec. 2021***. 46.9% of ***194*** countries have adopted policies authorizing HIV Self-Testing in-country, 50.0% have not adopted, and information is unavailable for 3.1% of countries.

Fig 2. Percent of ***194*** Countries by Adoption Status of Age of Consent Policies***, Dec. 2021***. 22.4% of ***194*** countries have adopted policies authorizing lowered age of consent to HIV testing and treatment services in-country, 57.7% have not adopted, and information is unavailable for 19.9% of countries.

Fig 3. Percent of ***194*** Countries by Adoption Status of PrEP Eligibility Policies***, Dec. 2021***. 27.6% of ***194*** countries have adopted policies authorizing PrEP approval in-country, 15.3% have partially adopted, 37.2% have not adopted, and information is unavailable for 19.9% of countries.”

Author Response: Thank you for pointing this out. We have clarified the number of total countries from which particular adoption statuses have been calculated to the figure titles, the figures themselves, and the captions. We have also added the year for this calculation to each of the figure titles, figures, and captions.

Comment: Is this the main result of the study? If there are more main results, they should be highlighted here in the first paragraph of the Discussion (Scientific Writing).

(Page 8, lines 169-171)

Original: “Adoption of policies supporting HIV self-testing, broad eligibility for PrEP, and lowered age of consent for HIV testing and treatment services all strongly correlated with PrEP uptake in-country.”

Revised: “Adoption of policies supporting HIV self-testing, broad eligibility for PrEP, and lowered age of consent for HIV testing and treatment services all strongly correlated with PrEP uptake in-country, ***with similar significance values. Yet only 13 of 194 countries have adopted all three policies, underscoring a missed opportunity to strengthen access to HIV prevention services.***”

Author Response: This is the main result of the study. However, we elaborated on it to emphasize why this result is important.

Comment: With this, it is concluded, in this paragraph, what from these findings compared to those of the study in question? Suggestion: contest…

(Page 10, line 224)

Original: “Additionally, while many countries have reported adoption of WHO PrEP recommendations into national guidelines, this does not necessarily translate into implementation of PrEP services or access to PrEP for all populations in need [31].”

Revised: “Additionally, while many countries have reported adoption of WHO PrEP recommendations into national guidelines, this does not necessarily translate into implementation of PrEP services or access to PrEP for all populations in need [31]. ***These findings enrich our analysis by clarifying that while policies are necessary for fostering enabling environments for HIV PrEP uptake, inequities in PrEP access despite the existence of enabling policies must also be addressed.***”

Author Response: Thank you for this suggestion. We have added a concluding sentence detailing how findings related to PrEP access disparities relate to our analysis. We have noted that policies that foster enabling environments for HIV PrEP uptake are necessary, but inequities in access to PrEP must also be addressed.

Comment: In contrast, what would be the potential of these findings? Suggestion: include as potentialities of this study.

(Page 11, line 247)

Original:“Additional limitations of this analysis include that we have used secondary sources for our research and that the data sources used for this analysis were missing data points for some countries.”

Revised: “Additional limitations of this analysis include that we have used secondary sources for our research and that the data sources used for this analysis were missing data points for some countries. ***Despite these limitations, our findings underscore that enabling policy environments can foster greater PrEP uptake. Governments and advocates can leverage this evidence to promote adoption of enabling policies for HIV prevention services including policies covered in this analysis, as a tool to address the HIV epidemic.***”

Author Response: Thank you for this suggestion. We have added the main potential of our findings and how it can be put into action by governments and advocates.

Comment: Suggestion: End the Discussion with this conclusion:

"250 While the adoption of PrEP recommendations in national guidelines is a vital step towards

251 increasing the availability of PrEP in the country, much more needs to be done to implement

252 PrEP services. With several additional PrEP options in the pipeline, equitable access will depend

258 a policy landscape that includes diverse PrEP choices and needs and breaks down barriers

259 to engage in HIV prevention services. Civil society plays a vital role in identifying the needs of

260 the most vulnerable and protecting their rights. Therefore, when considering future policy changes

261 or adoption of new PrEP options, governments and civil society must continue to

262 collaborate and strengthen joint efforts to effectively control the HIV epidemic."

(Page 11-12, line 250-262)

Original: “While ***adopting*** PrEP recommendations into national guidelines is a ***vital step*** towards increasing PrEP availability in-country, much more needs to be done to implement effective PrEP services. Despite the fact that policies permitting HIV self-testing, PrEP eligibility, and lowered age of consent to HIV testing and treatment are significantly correlated with PrEP uptake, many countries still do not have some or all of these policies in place. Results from this analysis imply that ***favorable policy environments*** have direct links to PrEP uptake and that policies facilitating access to HIV prevention services will be vital in effectively ***curbing*** the HIV epidemic. With ***multiple*** additional PrEP options in the pipeline, equitable access will depend on a policy landscape that ***is inclusive of diverse*** choices and PrEP needs and breaks down barriers to ***engaging*** in HIV prevention services. Civil society plays a vital role in identifying the needs of the most vulnerable and protecting their rights. Therefore, **in** considering future policy changes or adoption of new PrEP options, governments and civil society ***should*** continue to actively collaborate and strengthen joint efforts to effectively control the HIV epidemic.”

Revised: “While ***the adoption*** of PrEP recommendations into national guidelines ***exists and is a vital*** ***step*** towards increasing PrEP availability in-country, much more needs to be done to implement effective PrEP services. Despite the fact that policies permitting HIV self-testing, PrEP eligibility, and lowered age of consent for HIV testing and treatment are significantly correlated with PrEP uptake, many countries still do not have some or all of these policies in place. Results from this analysis imply that ***enabling policy environments*** have direct links to PrEP uptake and that policies facilitating access to HIV prevention services will be vital to effectively ***contain*** the HIV epidemic. With ***several*** additional PrEP options in the pipeline, equitable access will depend on a policy landscape that ***includes*** diverse PrEP choices and needs and breaks down barriers to ***engage*** in HIV prevention services. Civil society plays a vital role in identifying the needs of the most vulnerable and protecting their rights. Therefore, ***when*** considering future policy changes or adoption of new PrEP options, governments and civil society ***must*** continue to actively collaborate and strengthen joint efforts to effectively control the HIV epidemic.”

Author Response: This suggestion is well-taken. We have incorporated the majority of the changes into the Conclusion.

Comment: Suggestion: carry out an objective conclusion Suggestion: Conclusion

Despite the fact that policies that allow for HIV self-testing, eligibility for PrEP, and lowering the age of consent for HIV testing and treatment are significantly correlated with PrEP, many countries still do not have some or all of these policies in place. results of this imply that enabling policy environments have direct links to PrEP adoption and that policies that facilitate access to HIV prevention services will be vital to effectively contain HIV epidemic.

(Page 11-12, line 250-262)

Original: “While ***adopting*** PrEP recommendations into national guidelines is a ***vital step*** towards increasing PrEP availability in-country, much more needs to be done to implement effective PrEP services. Despite the fact that policies permitting HIV self-testing, PrEP eligibility, and lowered age of consent to HIV testing and treatment are significantly correlated with PrEP uptake, many countries still do not have some or all of these policies in place. Results from this analysis imply that ***favorable policy* *environments*** have direct links to PrEP uptake and that policies facilitating access to HIV prevention services will be vital in effectively ***curbing*** the HIV epidemic. With ***multiple*** additional PrEP options in the pipeline, equitable access will depend on a policy landscape that ***is inclusive of diverse*** choices and PrEP needs and breaks down barriers to ***engaging*** in HIV prevention services. Civil society plays a vital role in identifying the needs of the most vulnerable and protecting their rights. Therefore, **in** considering future policy changes or adoption of new PrEP options, governments and civil society **should** continue to actively collaborate and strengthen joint efforts to effectively control the HIV epidemic.”

Revised: “While ***the adoption*** of PrEP recommendations into national guidelines ***exists and is a vital*** ***step*** towards increasing PrEP availability in-country, much more needs to be done to implement effective PrEP services. Despite the fact that policies permitting HIV self-testing, PrEP eligibility, and lowered age of consent for HIV testing and treatment are significantly correlated with PrEP uptake, many countries still do not have some or all of these policies in place. Results from this analysis imply that ***enabling policy environments*** have direct links to PrEP uptake and that policies facilitating access to HIV prevention services will be vital to effectively ***contain*** the HIV epidemic. With ***several*** additional PrEP options in the pipeline, equitable access will depend on a policy landscape that ***includes*** diverse PrEP choices and needs and breaks down barriers to ***engage*** in HIV prevention services. Civil society plays a vital role in identifying the needs of the most vulnerable and protecting their rights. Therefore, ***when*** considering future policy changes or adoption of new PrEP options, governments and civil society ***must*** continue to actively collaborate and strengthen joint efforts to effectively control the HIV epidemic.”

Author Response: Thank you for this suggestion. We have accepted some of the changes to the relevant sentences. Please note that we kept “...correlated with PrEP uptake…” rather than “...correlated with PrEP…” to be specific that the focus was onPrEP uptake as it relates to policy adoption statuses. In this same vein, we kept the following unchanged: “...have direct links to PrEP uptake…”

Comment: Suggestion: include the word "exist"

(Page 11-12, line 250-262)

Original: “While adopting PrEP recommendations into ***national guidelines is*** a vital step towards increasing PrEP availability in-country, much more needs to be done to implement effective PrEP services.”

Revised: “While the adoption of PrEP recommendations into ***national guidelines exists*** and is a vital step towards increasing PrEP availability in-country, much more needs to be done to implement effective PrEP services.”

Author Response: We have added the word “exist” to the first sentence of the conclusion as indicated. The sentence is now: “While the adoption of PrEP recommendations into national guidelines exists and is a vital step towards increasing PrEP availability in-country, much more needs to be done to implement effective PrEP services.”

Comment: Suggestion: review references and include "DOI", if any.

(Page 12, line 268)

Author Response: Thank you for this suggestion. DOIs have been added to all reference citations where available.
